# Pleuroparenchymal fibroelastosis: role of high-resolution computed tomography (HRCT) and CT-guided transthoracic core lung biopsy

**DOI:** 10.1007/s13244-015-0448-3

**Published:** 2015-11-17

**Authors:** Cátia Esteves, Francisco R. Costa, Margarida T. Redondo, Conceição S. Moura, Susana Guimarães, António Morais, José M. Pereira

**Affiliations:** Department of Radiology, Centro Hospitalar de São João, Alameda Professor Hernâni Monteiro, 4200-319 Porto, Portugal; Department of Pneumology, Centro Hospitalar de São João, Alameda Professor Hernâni Monteiro, 4200-319 Porto, Portugal; Department of Pathology, Centro Hospitalar de São João, Alameda Professor Hernâni Monteiro, 4200-319 Porto, Portugal; Faculty of Medicine, University of Porto, Porto, Portugal

**Keywords:** Idiopathic interstitial pneumonia, Computed tomography, Biopsy, Lung, Fibrosis

## Abstract

**Objectives:**

Pleuroparenchymal fibroelastosis (PPFE) is a rare idiopathic interstitial pneumonia (IIP) with variable clinical and radiological features. Diagnosis is based on histology obtained by surgical lung biopsy, which is associated with significant mortality and morbidity. This study aims to briefly review PPFE and discuss the role of CT-guided transthoracic core lung biopsy (TTB) in its diagnosis.

**Materials/Methods:**

Four cases of PPFE diagnosed at our institution with TTB are reported and discussed.

**Results:**

Clinical, radiological and histological features are in agreement with the previous literature cases. TTB provided the diagnosis in all cases. Iatrogenic pneumothorax was the main complication in all patients. Placement of a chest tube was needed in three patients. An overlap between PPFE and other interstitial lung diseases (ILD) was documented.

**Conclusion:**

PPFE is an underdiagnosed IIP, so radiologist awareness of it needs to be widespread in patients with fibrosis with apical-caudal distribution. Coexistence of different lung diseases strengthens the idea of a predisposing factor. TTB proved to be a good diagnostic tool and can be considered the first choice for invasive assessment of these patients. PFFE has a variable course with no established therapeutic options; therefore a multidisciplinary team is crucial in the approach to patients with ILD.

**Main messages/Teaching Points:**

• *PPFE should be considered in the differential diagnosis of fibrosis with apical-caudal distribution.*

• *CT-guided TTB can be considered the first choice for invasive assessment of PPFE.*

• *Site of biopsy has to be chosen carefully in order not to miss PPFE.*

• *Coexistence of different lung diseases strengthens the idea of a predisposing factor.*

• *A multidisciplinary team is crucial in the approach to patients with ILD.*

## Introduction

Pleuroparenchymal fibroelastosis (PPFE) is a rare idiopathic interstitial pneumonia (IIP) first described as idiopathic pulmonary upper lobe fibrosis in 1992 in the Japanese literature by Aminati et al. [1] and with approximately 70 patients described in the English-language literature until now [1–11]. This entity is characterised by fibrotic and elastotic thickening of the pleura and subpleural lung parenchyma, mainly in the upper lobes [4]. Although the aetiology of PPFE is considered idiopathic in most cases, an association with a wide range of factors has been reported such as genetic predisposition; recurrent lower respiratory tract infections; underlying diseases or conditions such as collagen vascular diseases, bone-marrow transplantation or lung transplant patients suffering from restrictive allograft syndrome [5–8]. The therapeutic approach is highly variable in the published clinical cases with no clearly definitive therapeutic options other than lung transplantation [9]. Disease progression occurs in 60 % of patients with death from disease in 40 % [10]. The largest series of patients reported showed that of ten patients, five died during the first 2 years of evolution [1–11]. Nevertheless, no specific data related with survival is defined, but an overall bad prognosis can be assumed based on available data.

In all the published literature case series of PPFE the diagnosis was made by surgical lung biopsy [1–11]. However, the role of surgical biopsy in the diagnosis of IIP is still controversial because it can be associated with significant mortality and morbidity [12–15] with previous reports showing mortality rates between 0 and 17 % and a morbidity rate of 13 % [12]. On the other hand, Manhire et al. documented only a 0.15 % mortality rate with CT-guided transthoracic lung biopsy (TTB) [16] while some articles report an aetiological diagnosis in 79 to 100 % of the cases of interstitial lung diseases (ILDs) obtained by TTB [17–20].

The aim of this article is to review the clinical and imaging findings with pathological correlation of PPFE and to discuss the role of CT-guided transthoracic core lung biopsy (TTB) in the diagnosis of PPFE.

## Materials and methods

Four patients with PPFE underwent imaging evaluation at our institution between 2011 and 2014. High-resolution computer tomography (HRCT) was performed in all the patients. The medical records and radiology reports of all patients were reviewed and discussed in the Multidisciplinary Interstitial Lung Disease Board (MILDB) performed weekly (which includes pulmonologists, thoracic radiologists and pathologists). Each patient was posteriorly submitted to TTB in a dual-slice spiral CT scanner (Siemens Somatom® Emotion Duo) with fluoroscopy. An automatic core biopsy system (Bard® Magnum®) with an 18-gauge cutting needle (variable length, depending on the disease’s depth) was used in all patients. All the patients had local anaesthesia with 10 cc lidocaine hydrochloride 2 %. The biopsy was performed in the area of greater pleuroparenchymal thickness and the needle was placed outside the visceral/parietal pleura before performing the biopsy. Only one core biopsy was made in all patients. The samples obtained were preserved in a 10 % formaldehyde solution. A chest radiogram was posteriorly performed to evaluate complications. Histological samples were analysed and interpreted by two different pulmonary pathologists who agreed on the diagnosis in all cases.

## Results

### Clinical, laboratory and pulmonary tests features (Table 1)

All patients were females aged between 59 and 67 at presentation. Patient 4 had a history of smoking and patient 2 had a history of pulmonary tuberculosis, post-infectious bronchiectasis and recurrent respiratory infections. Patients 3 and 4 had occupational inhalation exposure: patient 3 has current environmental inhalation exposure (birds) and familiar history of interstitial lung disease (mother died of idiopathic pulmonary fibrosis); patient 4 had a previous diagnosis of chronic hypersensitivity pneumonitis.

**Table 1:** Clinical characteristics, pulmonary function test data and biopsy complications

Characteristics	Patient 1	Patient 2	Patient 3	Patient 4
Age at diagnosis (years)	67	59	64	60
Gender	Female	Female	Female	Female
Smoking status	Never smoker	Never smoker	Never smoker	Ex-smoker
Personal history	None	Pulmonary tuberculosis; post-infectious bronchiectasis; recurrent respiratory infections	Occupational exposure (varnishes, 2 years; paints and butyl acetate, 3 years) Exposure to birds at the diagnosis for 3 years	Occupational exposure (carpets and cork) Previous diagnosis of chronic hypersensitivity pneumonitis
Family history	None	None	Mother died of IPF^3^	None
Symptoms	DOE^2^, nonproductive cough	DOE^2^, productive cough	DOE^2^, nonproductive cough	DOE^2^, nonproductive cough
Pulmonary function data	Mild restrictive ventilatory impairment with mild decreased of diffusion capacity	Moderate restrictive ventilatory impairment with severe decrease of diffusion capacity	Mild restrictive ventilatory impairment with mild decreased of diffusion capacity	Mild restrictive ventilatory impairment with mild decreased of diffusion capacity
Biopsy complications				
1. Subcutaneous emphysema	Yes	No	No	No
2. Pneumothorax	Yes	Yes	Yes	Yes
CTP^1^	Yes	Yes	Yes	No
Length of hospital stay	17 days (1st biopsy inconclusive; the two biopsies were performed with a 10-day interval)	5 days	3 days	1 day
Follow-up	Died - 20 months after diagnosis	Alive - 36 months after diagnosis	Alive - 24 months after diagnosis	Alive - 18 months after diagnosis

^1^CTP - chest tube placement, ^2^DOE - dyspnoea on exertion, ^3^IPF - idiopathic pulmonary fibrosis

At presentation all patients complained of dyspnoea on exertion with progressive worsening and cough. Bronchoalveolar lavage and bronchoscopy indicated no abnormalities and bronchial biopsies showed no evidence of malignancy, granulomas or other diagnoses.

Lung function tests showed a mild restrictive ventilatory impairment (CVF 74.23 ± 13.5) and mild decrease of diffusion capacity (DLCO 53.1 ± 17.05). All patients demonstrated oxygen desaturation in the 6-min walking test. Bronchoalveolar lavage was performed in three patients. Two of them had neutrophilia and eosinophilia, and the other had lymphocytosis and eosinophilia.

### Imaging and biopsy findings

**Fig. 1:**
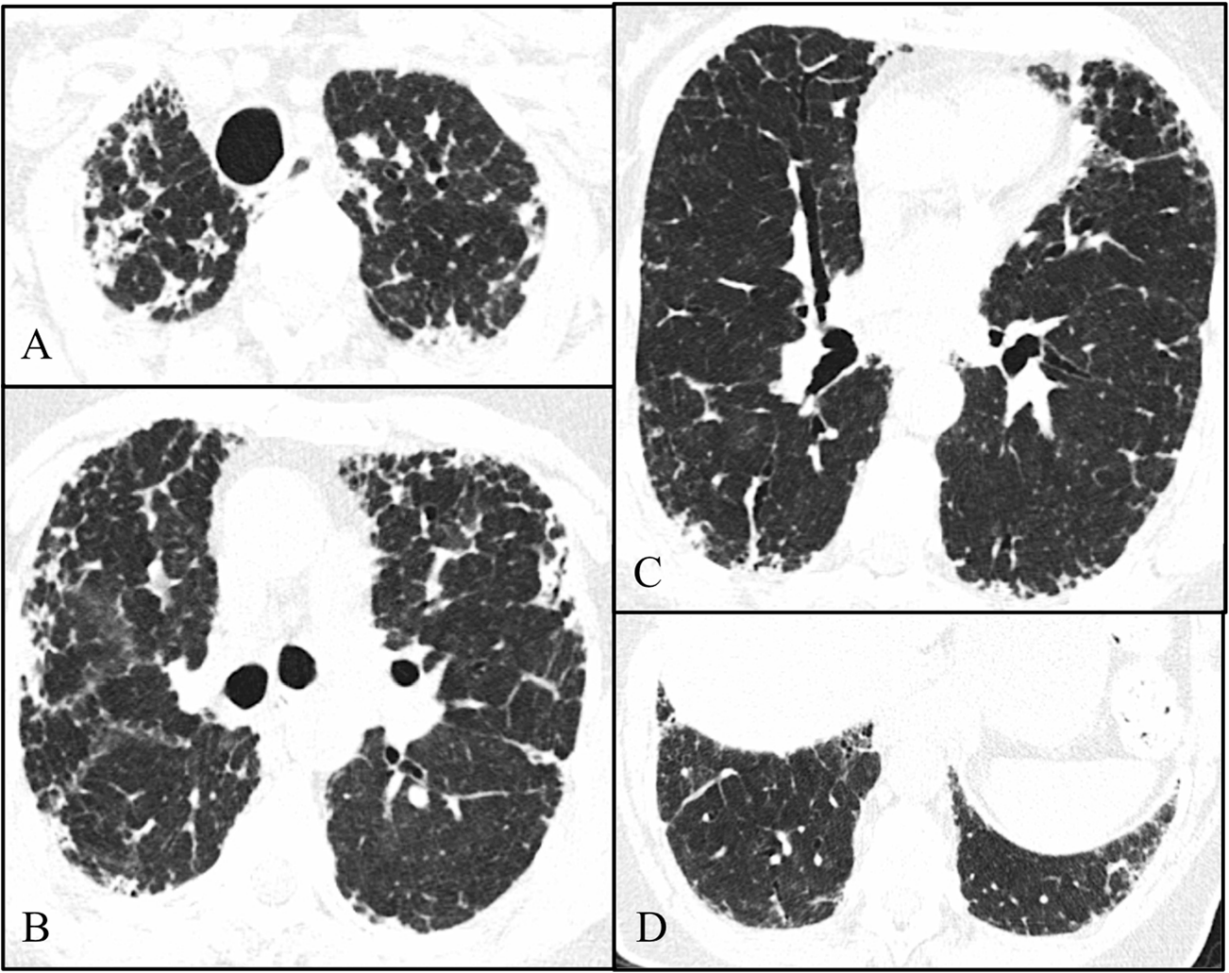
Axial high-resolution chest tomography (HRCT) images (**a**–**c**) and coronal reformatted images (**d**) of patient 1 show pleural and subpleural thickening with severe fibrotic changes in the marginal parenchyma with apical-caudal distribution. Some areas of traction honeycombing are also seen

**Fig. 2:**
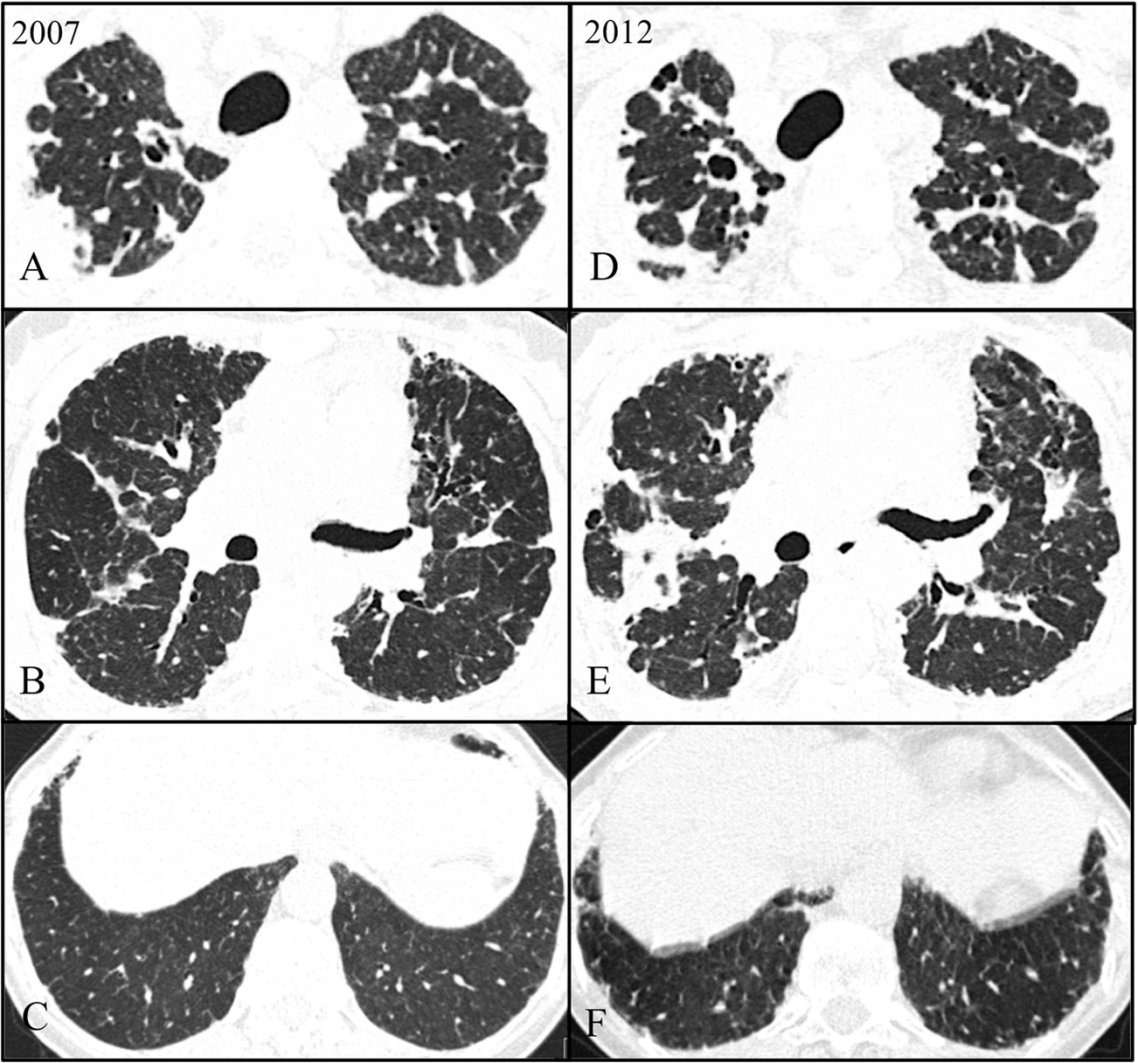
Axial high-resolution chest tomography (HRCT) images in 2007 (**a**–**c**) and 2012 (**d**–**f**) of patient 2 show pleural and subpleural thickening with severe fibrotic changes in the marginal parenchyma with apical-caudal distribution. Some areas of traction bronchiectasis and honeycombing are also seen. Note the evolution between 2007 and 2012 (at the diagnosis)

**Fig. 3:**
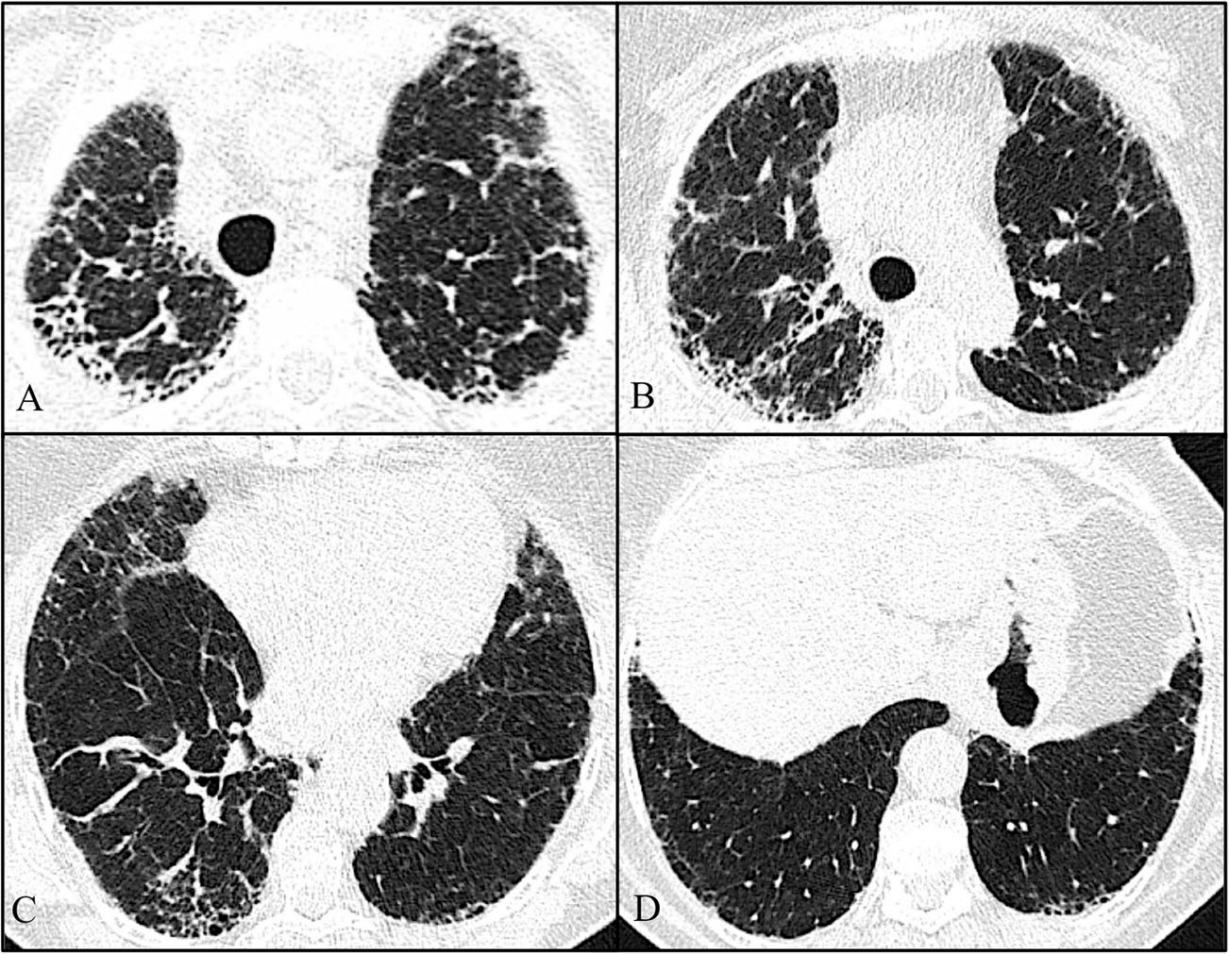
Axial high-resolution chest tomography (HRCT) images (**a**–**c**) and coronal reformatted images (**d**) of patient 3 show pleural and subpleural thickening with moderate fibrotic changes in the marginal parenchyma with apical-caudal distribution. Some areas of honeycombing are also seen

**Fig. 4:**
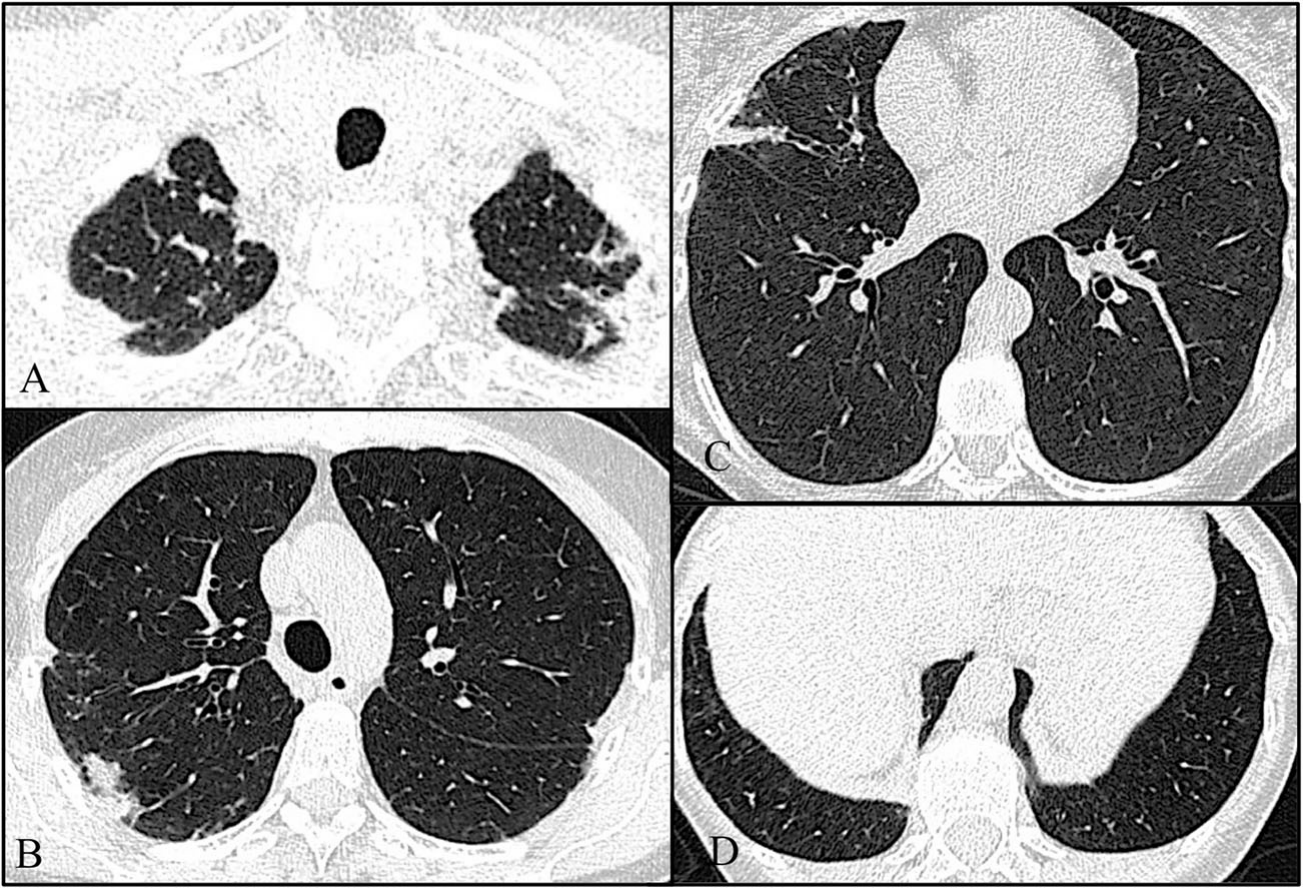
Axial high-resolution chest tomography (HRCT) images (**a**–**d**) of patient 4 show pleural and subpleural thickening with moderate fibrotic changes in the marginal parenchyma with apical-caudal distribution. These features are more evident in the right lobe. Note also, in **c**, a slight “tree-in-bud” pattern in the middle lobe compatible with concomitant pulmonary infection

HRCT images show dense pleural and subpleural consolidation with a reticular pattern, predominantly in the upper lobes. The upper lobes were always more severely involved, with involvement of the lower lobes being absent or less marked (apical-caudal distribution). Associated features included upper lobe volume loss, architectural distortion and traction bronchiectasis (Figs. 1, 2, 3 and 4). One of the patients showed significant worsening relative to previous examinations (Fig. 2).

**Fig. 5:**
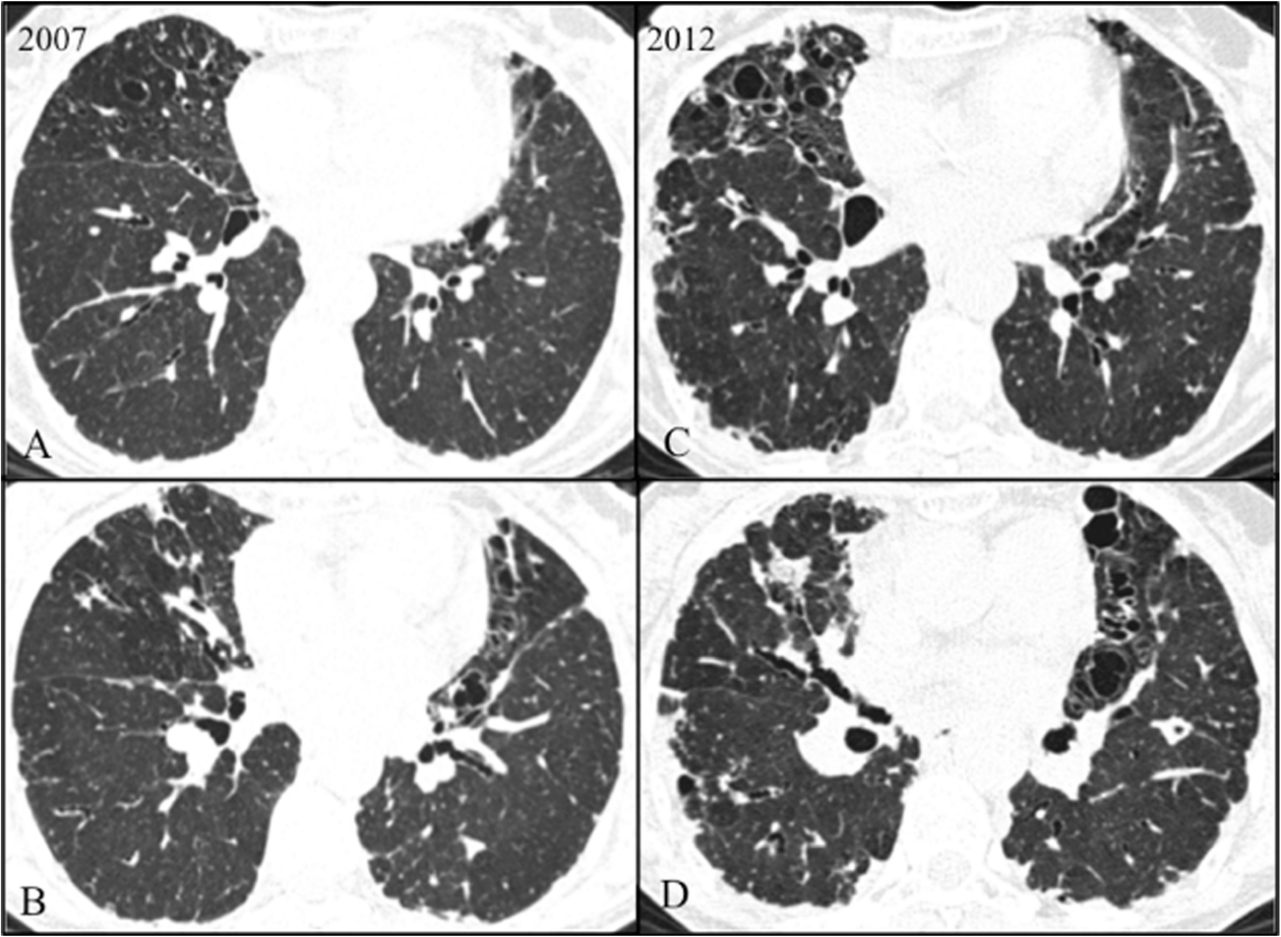
Axial HRCT scans in 2007 (**a**–**b**) and 2012 (**c**–**d**) in patient 2 show multiple cystic bronchiectasis in the middle right lobe and lingula with worsening between 2007 and 2012

**Fig. 6:**
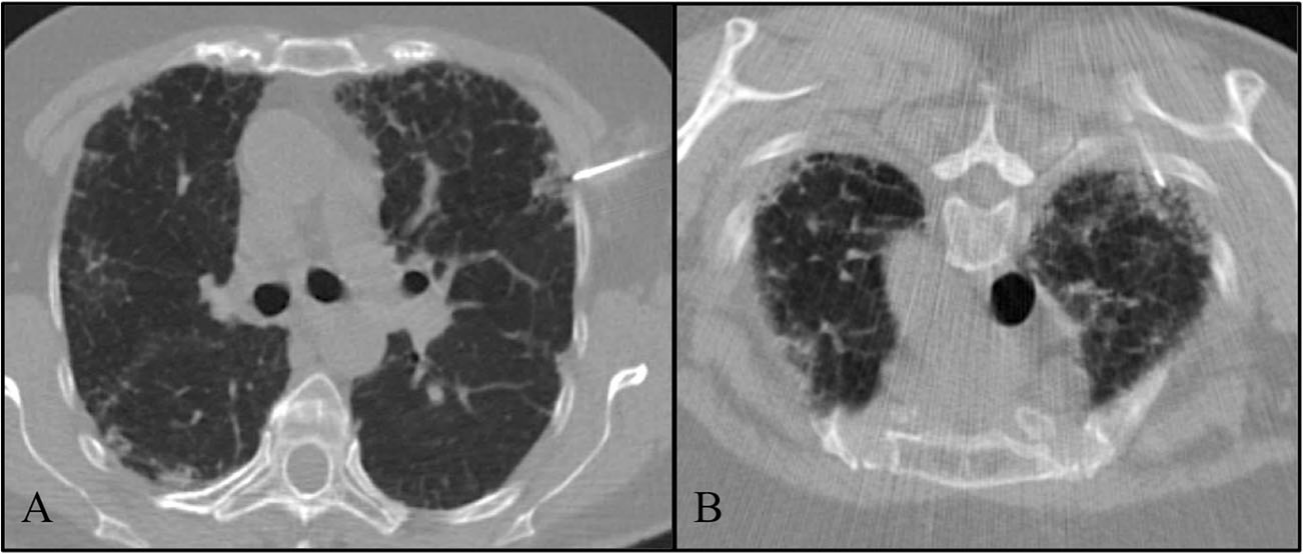
Ancillary image of a CT-guided transthoracic core lung biopsy. TTB at the upper left lobe in patient 1 (**a**) and TTB in patient 3 at the upper right lobe (**b**)

Two of the patients showed reticular opacities with some areas of honeycombing and traction bronchiectasis (Figs. 1, 2 and 3). The other patient had co-existent cystic bronchiectasis (Fig. 5). The patients underwent a TTB in the higher density zones (Fig. 6). All the patients were complicated with mild to moderate iatrogenic pneumothoraxes. Placement of a chest tube was needed in three patients. The pneumothorax resolved completely before discharge with a hospital stay ranging between 1 day and 17 days. One of the patients was also complicated with subcutaneous emphysema.

### Pathological findings

**Fig. 7:**
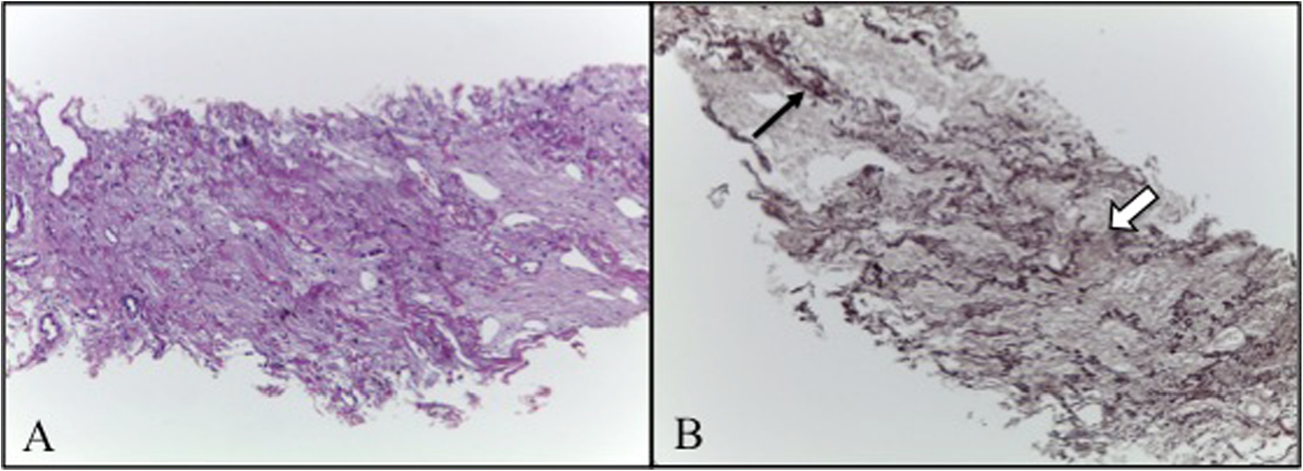
**a** Thickened visceral pleura and prominent subpleural fibrosis characterised by abnormal increase of elastic tissue and dense collagen (H&E, 100×). Abrupt transition to normal parenchyma is also seen. Parenchyma distant from the pleura is spared. **b** Elastosis of the alveolar walls (*black arrow*; orcein, 100×.) with a predominant intra-alveolar fibrosis (*white arrow*)

Histological evaluation showed, in all four cases, pleural and subpleural fibrosis with an abrupt transition to normal lung parenchyma. The elastic stains demonstrate deposition of dense elastic fibers (elastosis) in the subpleural fibrotic lung lesion and alveolar walls confirming the diagnosis (Fig. 7).

### Follow-up

Only one of the patients clinically and functionally worsened after 20 months of follow-up and died because of nosocomial pneumonia after a surgery for hiatal hernia correction (patient 1). The other patients have been clinically and functionally stable until now.

## Discussion

PPFE is a rare, underdiagnosed and increasingly recognised disorder with unique clinical, radiological and pathological features [1–11, 21]. It has been defined as a distinct IIP entity in the English literature by Frankel et al. [5] and was recently included in the International Multidisciplinary Classification of the Idiopathic Interstitial Pneumonias of the American Thoracic Society/European Respiratory Society in the category of rare IIP [9].

Given its rarity in addition to the fact that it is a very recently recognised entity, with fewer than a hundred cases described until now, there are no reliable data on the incidence and prevalence [1–11].

PPFE also has a distinct therapeutic approach and prognosis in comparison to other IIPs; therefore an accurate diagnosis is important. Although there are no specific therapeutics with proven efficacy, the majority of patients described had been treated with immunosuppressant therapy and a minority associated with recurrent infections with plaquinol. This is significantly different from other IIPs such as idiopathic pulmonary fibrosis treated with antifibrotics such as pirfenidone or nintedanib or others with a predominant inflammatory component treated mainly with corticoids [1–11].

In the majority of the patients reported in the literature the clinical course was progressive, despite aggressive treatment. The survival however is variable, ranging from 4 months to 7 years following diagnosis, with half of the patients dying in the first 2 years. Therefore, early diagnosis is essential [1–11].

PPFE presents in adults with a median age of 57 years with no sex predilection and occurs most commonly in patients with absence of smoking habits [12]. In our data the patients were older (median age of 62.5 years) and all were female. Three patients were non-smokers, being in line with previous studies. Concerning the aetiology, our data are also in agreement with published cases with one of the patients having a history of recurrent respiratory infections and other familiar history of IIP [12].

There is also a certain variability of clinical presentation and the symptoms are often nonspecific with the most common presentation symptoms published being shortness of breath and cough, also in line with our cases [1–12]. HRCT findings in all cases were in agreement with the previously described series and the histopathological features obtained by TTB were considered representative and compatible with the diagnosis of PPFE after a multidisciplinary discussion.

The most common complication with TTB is pneumothorax, which occurs in 0–61 % of lung biopsies. Although rare, other complications include pulmonary haemorrhage, haemothorax, gas embolism and cardiorespiratory arrest [16, 20]. In the case series of PPFE, the published patients were submitted to surgical lung biopsy [1–12] with a reported death of one patient following surgical lung biopsy complicated by large bronchopleural fistulae [11]. Becker et al. also postulated that these patients might be prone to the development of secondary spontaneous pneumothoraxes [11]. In our study the diagnosis of PPFE was made by TTB in all patients. Although all patients had pneumothoraxes, they resolved before discharge with only one patient needing hospitalisation for more than 1 week. This patient needed two biopsies, was the first diagnosed at our institution and had the worst prognosis, dying 20 months after diagnosis. Therefore, TTB can be considered the first choice for the invasive assessment of PPFE, sparing patients a late surgical lung biopsy. However, we only had four patients, and more reports are needed to improve the reliability.

Chronic hypersensitivity pneumonitis and apical caps are differential diagnoses to consider when there is predominant involvement of the upper lobes, and they can have radiological and histological features that overlap with those of PPFE. The latter is particularly challenging, especially when associated with tuberculosis scars, which, in our country, have a medium incidence. Apical caps also tend to occur in older cigarette smokers and are localised in the upper lobes, usually with no extension to other lobes. In these patients centrilobular emphysema can be seen and they are typically asymptomatic with nonprogressive disease [4, 5]. On the other hand, PPFE has a more diffuse subpleural distribution and can also affect basal portions and other lobes [4, 5]. In the case of hypersensitivity pneumonitis like Piciucchi et al. described, lesions have a bronchiolar location and do not have a subpleural and pleural predominance like in PPFE [4]. In our study one patient had a history of previous tuberculosis and two other environmental exposures, making the diagnosis more challenging. One of them even had a misdiagnosis of chronic hypersensitivity pneumonitis. Therefore, PPFE must be considered in the differential diagnosis of these entities to alert pathologists to perform elastic fibre stains, making the diagnosis easier.

Reddy et al. also describe a high prevalence of coexistent ILDs, such as nonspecific interstitial pneumonia (NSIP) and usual interstitial pneumonia (UIP). They describe a pattern of ILD in 5 of 12 patients with PPFE and bronchiectasis in 1 of the patients. The features of coexistent fibrosis, including UIP, have been described more extensively in the Japanese literature [7]. Our study, like in previous reports, shows that three patients had other concomitant pleuroparenchymal changes supporting the previously postulated concept that these patients might have a predisposition to lung diseases, which may manifest as different histopathological patterns of fibrosis [7]. Therefore, the location of the TTB needs to be carefully selected according to the HRCT to collect biopsies of PPFE and not from the coexistent lung disease.

Regarding follow-up, there are no guidelines related to PPFE follow-up, namely in a particular situation where a definitive diagnosis is not possible. However it is reasonable to propose lung function tests every 3–6 months, a thoracic x-ray every 6 months and a thoracic HRCT scan every 12 months. A close follow-up should be carried out for patients with a family history of PPFE who tend to have a more aggressive disease course and should have a close follow-up [3].

In conclusion, PPFE is an underdiagnosed disease, with uncertain prevalence and incidence. Thus, radiologists’ awareness of the condition needs to be widespread and routinely considered in patients with fibrosis with apical-caudal distribution to alert the pathologist to perform elastic fibre stains, making the diagnosis easier. The fact that a number of patients with PPFE demonstrated features of coexistent lung disease should also alert radiologists to consider both diffuse forms of PPFE and other lung diseases in the differential diagnosis. TTB has been shown to be a good diagnostic tool and can be considered the first choice for the invasive assessment of PPFE, sparing patients a late surgical lung biopsy. The biopsy site has to be chosen carefully according to the HRCT in order not to miss PPFE because of other lung diseases. This disease has a variable course with survival rates ranging from months to years, with no defined therapeutic options. Therefore, a Multidisciplinary Interstitial Lung Disease Board is crucial to the early diagnosis of PPFE as well as to establishing an adequate approach in patients with ILD.
